# Lateral meniscus repair contributes to controlling pivot shift in patients undergoing anterior cruciate ligament reconstruction with steeper lateral posterior tibial slope

**DOI:** 10.1002/jeo2.70717

**Published:** 2026-04-14

**Authors:** Masaki Amemiya, Yusuke Nakagawa, Tomomasa Nakamura, Nobutake Ozeki, Takashi Hoshino, Mai Katakura, Aritoshi Yoshihara, Ichiro Sekiya, Hideyuki Koga

**Affiliations:** ^1^ Department of Joint Surgery and Sports Medicine, Graduate School of Medical and Dental Sciences Institute of Science Tokyo Tokyo Japan; ^2^ Department of Cartilage Regeneration, Graduate School of Medical and Dental Sciences Institute of Science Tokyo Tokyo Japan; ^3^ Center for Stem Cell and Regenerative Medicine Institute of Science Tokyo Tokyo Japan

**Keywords:** anterior cruciate ligament, anterolateral rotatory instability, lateral meniscus repair, lateral posterior tibial slope

## Abstract

**Purpose:**

The lateral meniscus (LM) controls anterolateral rotatory instability (ALRI) and is considered a secondary restraint. However, how the anterior cruciate ligament (ACL) and LM contribute to ALRI control remains unclear. This study investigated the degree to which ACL and LM contribute to ALRI control and examined factors contributing to ALRI during anterior cruciate ligament reconstruction (ACLR).

**Methods:**

Twenty‐six patients in ACL and LM‐injured knees were enroled. Tibial acceleration in pivot shift was measured using a triaxial accelerometer by adjusting ACL graft tension before and after LM repair. ACL graft tension required for below‐healthy‐side pivot shift acceleration (minimum required tension [MRT]) was measured before and after LM repair. Patient factors for a larger contribution of LM to ALRI were determined by univariate linear regression analysis between changes in pivot shift acceleration before and after LM repair, and explanatory variables included patient and morphological factors. Furthermore, multiple regression analysis was conducted to identify independent factors significantly associated with the change.

**Results:**

LM repair reduced the pivot shift acceleration in most cases, even following ACLR fixed at 10 N for each bundle (20 N in total) and pivot shift acceleration decreased significantly from 4.3 ± 1.2 to 2.9 ± 0.9 m/s^2^ by LM repair (*p* < 0.001). The MRT was 10 N without LM repair in 13 patients (50%). LM repair reduced MRT to 10 N in 11 of the remaining 13 patients. Only the lateral posterior tibial slope (PTS) significantly correlated with changes in pivot shift acceleration (*r* = 0.60, *p* = 0.001). In the multiple regression analysis as well, lateral PTS was the only factor identified as a significant predictor.

**Conclusions:**

LM repair decreased pivot shift acceleration and MRT, indicating the importance of LM repair in force‐sharing with the ACL. LM contributes significantly to ALRI in patients with a larger lateral PTS. During ACLR, the LM should be repaired as much as possible.

**Level of Evidence:**

Level III, diagnostic study.

**Abbreviations**
ACL: anterior cruciate ligament
ACLR: anterior cruciate ligament reconstruction
ALRI: anterolateral rotatory instability
AMB: anteromedial bundle
BMI: body mass index
LM: lateral meniscus
LMPRT: lateral meniscus posterior root tear
MM: medial meniscus
MRI: magnetic resonance imaging
MRT: minimum required tension
PLB: posterolateral bundle
PTS: posterior tibial slope

## INTRODUCTION

Residual anterolateral rotatory instability (ALRI) after anterior cruciate ligament reconstruction (ACLR) has been reported to be 8%–25% [4, 14]. Several studies have reported that ALRI is associated with lower patient satisfaction, lower functional outcomes and progression of knee osteoarthritis [12, 34]. Thus, it is important to avoid residual ALRI subsequent to ACLR to improve clinical outcomes.

Accumulating evidence highlights the lateral meniscus (LM) as a critical secondary restraint against ALRI [7, 9, 11, 21, 24]. Previous studies have demonstrated that LM injuries, particularly LM posterior root tears (LMPRTs), can exacerbate ALRI, whereas LM repair significantly decreases the pivot shift in ACL‐deficient knees [7, 11, 21, 24]. Furthermore, LM is considered to remain a critical stabilizer even after ACLR. Hoshino et al. [9] reported a residual pivot shift after ACLR patients who underwent LM resection. However, it remains unclear to what extent LM contributes to the control of ALRI after anatomic ACLR, particularly when ACL graft tension is appropriately controlled. Because the ACL is the primary restraint against ALRI, control of the pivot shift depends not only on the presence of the reconstructed ACL but also on the amount of tension applied to the graft. Therefore, if the LM contributes to rotational control, successful meniscal repair may reduce the amount of tension required in the ACL graft to suppress the pivot shift phenomenon.

In addition to meniscus injury, other factors that have been reported to influence ALRI include younger age [10], knee hyperextension [10, 29, 33], generalized joint laxity [18], preoperative high‐grade pivot shift [10, 15, 33], time from injury to surgery [15] and anterior stability [5]. Recently, the relationship between ALRI and the bone morphology of the distal femur and the posterior tibial slope (PTS) has been described [5, 27, 28, 29]. However, it remains unclear whether LM repair influences ALRI in patients with these risk factors during ACLR.

Several clinical questions remain to be clarified. Specifically, it remains unclear to what extent the LM contributes to ALRI following technically sound ACLR, and whether this contribution is consistent across all clinical cases. This study aimed to investigate how ACL and LM contribute to controlling ALRI. Particularly, the tension in the ACL graft required to control pivot shift before and after LM repair was compared during ACLR. Furthermore, patient factors, including demographic data and bone morphology, that had a significant effect on ALRI resulting from LM repair were investigated by examining the correlation between the change in pivot shift grade due to LM repair and patient factors. It was hypothesized that LM repair would result in further control of ALRI following ACLR in a substantial proportion of patients and that patients with a larger lateral PTS would be more effective in decreasing ALRI with LM repair.

## MATERIALS AND METHODS

### Patients' enrolment

This study was approved by the Institutional Review Board of Tokyo Medical and Dental University (protocol number: M2000‐1566). All patients provided written informed consent to participate in the study. Patients with ACL injuries scheduled to undergo primary double‐bundle ACLR using an autologous semitendinosus tendon between June 2018 and April 2021 were included. Of these, cases of LM injury confirmed during the operation were included in the analyses. Exclusion criteria included concomitant ligament injuries, history of knee injuries in both knees, open physes at the time of surgery and osteoarthritis greater than Kellgren–Lawrence Grade 2. Patients with open physes were considered skeletally immature and were excluded from the study, as their typically smaller graft diameters could necessitate single‐bundle reconstruction, potentially compromising the uniformity of the ACL reconstruction procedure.

### Measurement protocol

**Figure 1:**
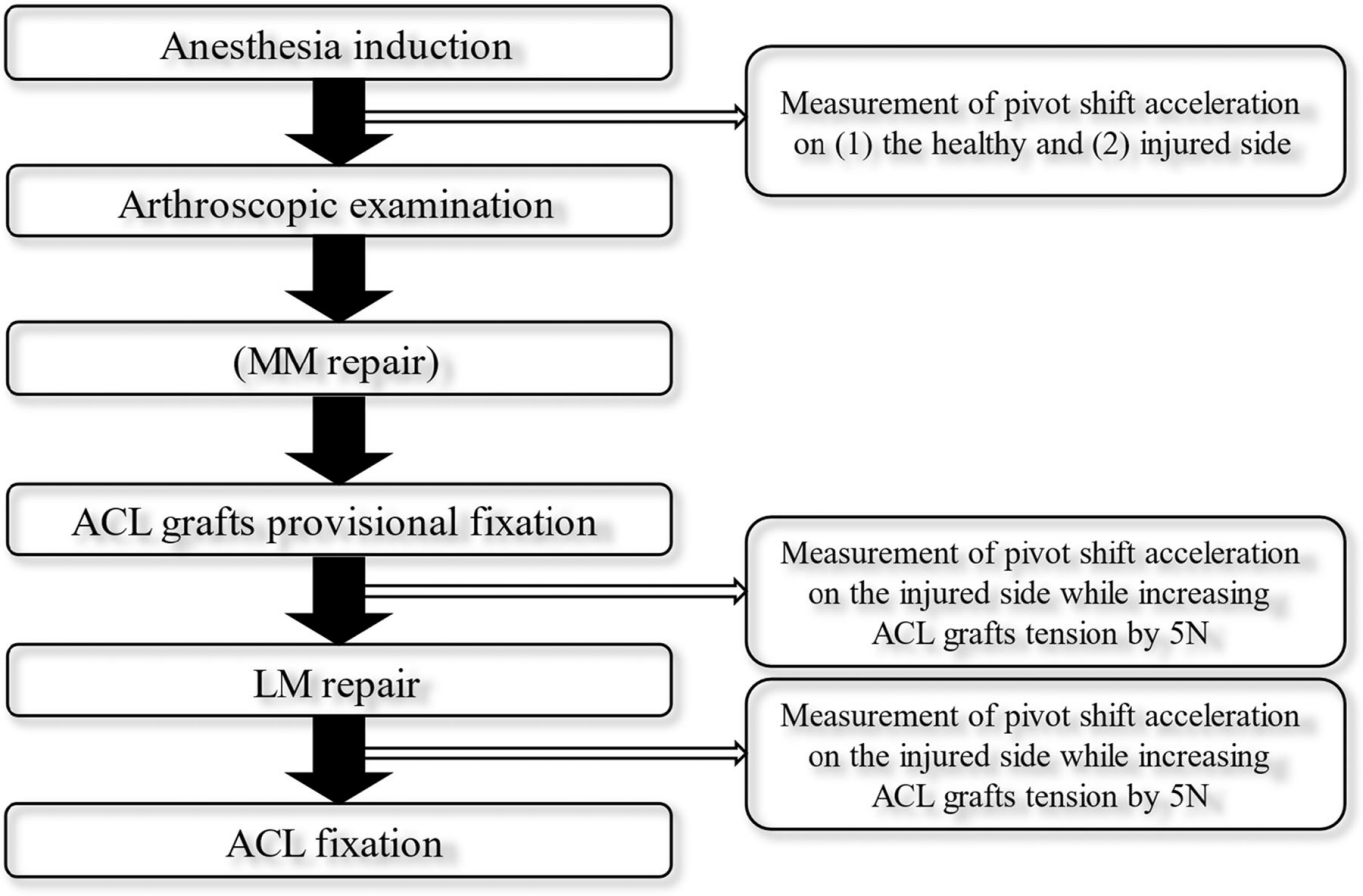
Scheme showing the measurement protocol. The acceleration during the pivot shift on the healthy and injured sides under anaesthesia was measured using KiRA. Torn ACL and LM were confirmed arthroscopically, and MM injury was treated first. After passing the ACL graft, it was provisionally fixed at 10 N, and the pivot shift acceleration was measured. The tension was then increased by 5 N and incremented until it was just below that of the healthy side. The LM was then repaired, and the measurements were performed in the same manner. ACL, anterior cruciate ligament; LM, lateral meniscus; MM, medial meniscus.

The quantitative evaluation of ALRI was performed using the pivot shift test with a kinetic rapid assessment (KiRA) triaxial accelerometer (Orthokey) (Figure 1). The reliability of this device has been previously reported [3, 25, 37, 38]. The device was fixed to the anterolateral aspect of the proximal tibia, between the tibial tuberosity and the Gerdy tubercle, using a belt. This tool measures the acceleration of the tibia during the pivot‐shift test (m/s^2^). The tool extracts the difference between the maximum value (*a*_max_) and the minimum value of acceleration (*a*_min_) (*a*_range_: *a*_max _− *a*_min_), to indicate the magnitude of subluxation during the pivot shift phenomenon, and the acceleration range was defined as ‘acceleration’ in the following analyses.

The pivot shift test was performed preoperatively by an attending surgeon under anaesthesia in injured and healthy knees. The pivot shift test was performed five times, and the acceleration was measured for each test. Subsequently, the maximum and minimum values were excluded, and the remaining three values were averaged.

Once ruptured ACL and torn LM were arthroscopically confirmed, the medial meniscal (MM) injury was treated according to the injury status if it existed. Next, bone tunnels were created, ACL grafts were inserted, and tibial acceleration was measured by performing the pivot shift test, whereas the grafts were provisionally fixed to the Graft Tensioning System (ConMed Linvatec) with a force of 10 N for both the anteromedial bundle (AMB) and posterolateral bundle (PLB) at a 20° knee flexion angle. The tension of the grafts increased by 5 N until the acceleration decreased below that of the healthy side. A minimum required tension (MRT) was defined as the lowest tension at which the pivot shift acceleration became below that of the healthy side. Then, LM was repaired according to the injury status, and the same measurements were repeated to measure pivot shift acceleration and to evaluate the MRT.

### Surgical procedure

All ACLRs were performed by seven experienced knee surgeons, each with over 10 years of clinical experience and at least 2 years of knee surgery training, under the supervision of two attending surgeons. Only the semitendinosus tendon was harvested using an open‐loop stripper. The harvested tendon was cut into halves and folded, creating two double‐stranded bundles of 5.5 cm or more in length, looped with EndoButton CL‐BTB (Smith & Nephew Endoscopy). Standard arthroscopic examination was performed through the anteromedial and anterolateral portals. Ruptured ACL and torn LM were confirmed arthroscopically, and the MM injury was treated according to the state of the injury.

Femoral tunnel creation was performed without removing the remnant tissue using the behind‐remnant approach in double‐bundle ACLR [16, 23]. Both femoral and tibial tunnels were created at the anatomic position of the insertion sites of each bundle. Femoral tunnel creation was made via the outside‐in approach, and tibial tunnel creation was made from the anteromedial surface of the tibia. Both grafts were inserted and fixed using EndoButton CL‐BTB for femoral fixation. For the pivot shift measurements, each graft was provisionally fixed to the Graft Tensioning System (Linvatec) with sutures at the tibial site in each graft fixation setting, as previously described. After all the measurements were finished, the tibial ends of the grafts were fixed using the TensionLoc implant system (Arthrex) at 20° of flexion. The tension applied to each graft was adjusted such that both bundles had the same tension, determined by the tension at which the ALRI was below that of the healthy side in the intraoperative measurement of the acceleration during the pivot shift test.

LM repair was performed after pivot shift measurements for ACLR alone, according to the pattern and location of the meniscal tears. LM tears confined to the posterior segment were sutured using the all‐inside technique, whereas LM tears extending to the middle segment were sutured using the inside‐out technique using the Henning system for inside‐out meniscal repair (Stryker Japan). For LMPRT, a pull‐out repair was performed by creating a bone tunnel at the attachment site using the ACUFEX Director Tip Aimer (Smith & Nephew Endoscopy), followed by a vertical mattress suture using 2‐0 Fiber Wire (Arthrex) and two racking hitch knots with suture tape applied to the torn edge of the LMPRT. Subsequently, the sutures were pulled through the tibial tunnel and were fixed using a TensionLoc implant system (Arthrex).

### Factors determining a greater contribution of LM to ALRI

To evaluate morphological factors, both radiographic and magnetic resonance imaging (MRI) assessments were performed. The medial and lateral femoral condylar widths were measured on coronal MRI images [29] and expressed as a percentage of the total femoral width. The distal femoral condyle offset was measured on lateral radiographs [5], and similarly expressed as a percentage. Medial and lateral PTS were measured using sagittal MRI of the knee [36]. To identify patient‐related factors associated with a greater contribution of LM to ALRI, a univariate linear regression analysis was conducted. The dependent variable was the change in pivot shift acceleration before and after LM repair, with ACLR tension fixed at 10 N for each bundle. Explanatory variables included age at the time of surgery, body mass index (BMI), range of motion of both knees, KT‐1000 arthrometer (side‐to‐side difference and values for healthy side), pivot shift acceleration of both knees, absence of MM injury, medial and lateral femoral condylar width, medial and lateral PTS and distal femoral condyle offset.

### Statistical analysis

Statistical analyses were performed using SPSS software (v.24.0; SPSS). The Wilcoxon signed‐rank test was used to compare the changes in pivot shift acceleration before and after LM repair. The Spearman's rank correlation coefficient was used to assess the correlation between the objective and explanatory variables. Explanatory variables having a *p* value < 0.2 were included in a multiple regression analysis. Multiple regression analysis was performed to assess the effect of explanatory variables on changes in pivot shift acceleration before and after LM repair. For all analyses, *p* < 0.05 was considered statistically significant. A priori power analysis was performed using G‐power 3.1 software (Kiel University). Based on a previous study [11], the effect size of the change in pivot shift acceleration before and after LM repair was approximately 0.80. Overall, 11 patients were required as the minimum sample size to obtain a power of 0.80 at an alpha level of 0.05. Based on a pilot study, it was assumed that half of the patients had controlled ALRI with ACLR; thus, a minimum of 22 patients would be required in this study. Post hoc power analysis of the change in tibial acceleration before and after LM repair following ACLR revealed that, with an alpha of 0.05, a sample size of 26 patients achieved a power of 0.99. The intra‐rater reliability for the pivot shift acceleration measurements was assessed using the intraclass correlation coefficient with all acceleration data. The intraclass correlation coefficient was 0.78.

## RESULTS

### Patient characteristics

After applying the inclusion and exclusion criteria, 26 patients were included in the study. Patient demographic data are shown in Table 1. The mean age at the time of surgery was 26 years (range, 15–50 years), and included 13 males and 13 females. The average preoperative period was 8 months (range, 1–62 months), and no patient presented with hyperextension >10°. The preoperative KT‐1000 arthrometer values were 8.4 mm (range, 5.0–13.0 mm) for the healthy side and 6.4 mm (range, 3.0–10.0 mm) for the side‐to‐side difference. MM injuries were observed in 14 patients; of these, 12 underwent meniscal repair. The all‐inside technique was the most commonly used suture method for LM (16/26), followed by pull‐out repair (7/26).

**Table 1:** Demographic data of the patients.

Patient demographics (*n* = 26)		
Age, years		21 (15–50)
Gender female/male, *n*		13/13
Beighton score		0 (0–4)
Preoperative period, months		3 (1–62)
ROM (extension), °		4 (0–8)
ROM (flexion), °		155 (140–155)
KT‐1000 (absolute value of healthy side), mm		8.25 (5.0–13.0)
KT‐1000 (side‐to‐side difference), mm		6.25 (3.0–10.0)
MM injury, *n*		14 (Repair 12)
LM repair, *n*		
All‐inside		16
Inside‐out		1
All‐inside + inside‐out		2
Pull‐out		7
Medial condyle width, %		34.9 (31.9–37.9)
Lateral condyle width, %		44.4 (38.8–47.6)
Medial posterior tibial slope, °		5.1 (2.1–7.7)
Lateral posterior tibial slope, °		4.6 (2.1–12.3)
Distal femoral condyle offset, %		65.0 (62.2–68.4)

*Note*: Values are expressed as median (range).

Abbreviations: LM, lateral meniscus; MM, medial meniscus; ROM, range of motion.

### Change in pivot shift acceleration

**Figure 2:**
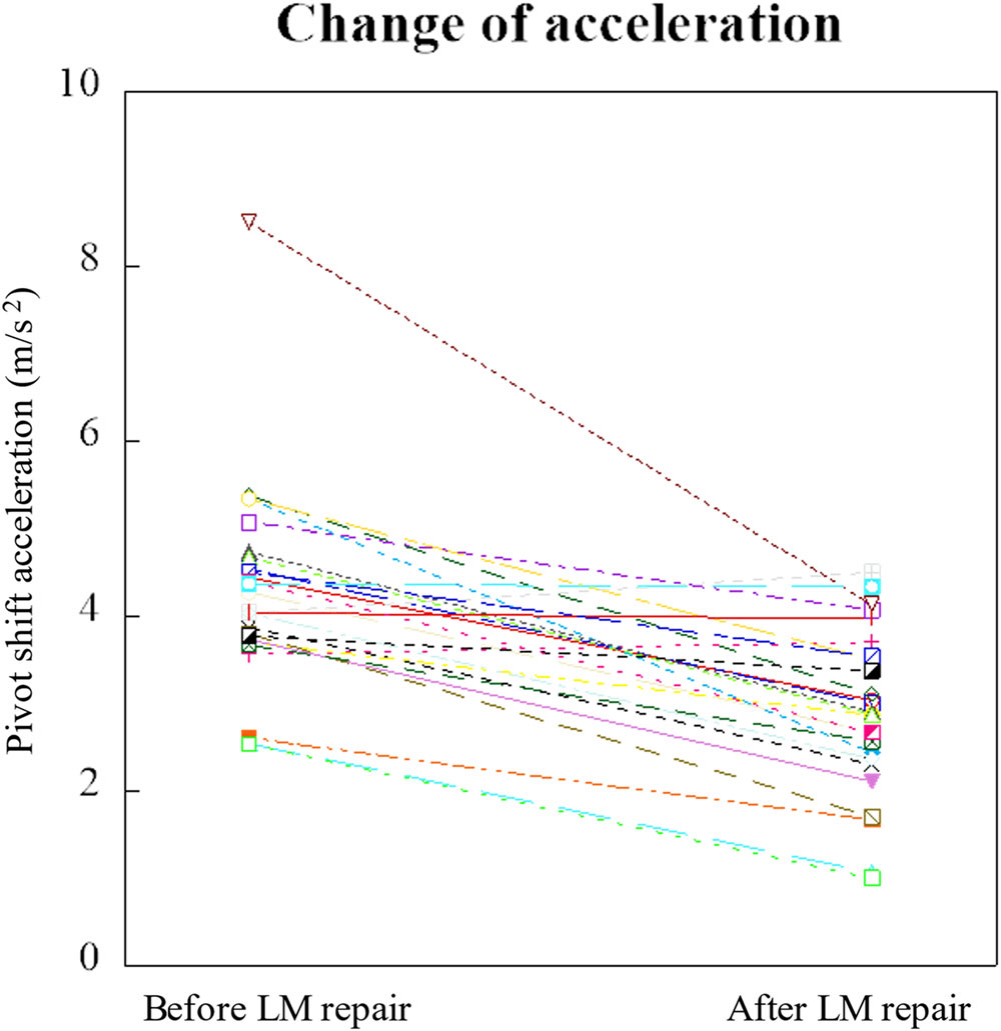
Change in pivot shift acceleration before and after LM repair under ACLR at 20 N (10 N each). LM repair reduced pivot shift acceleration in most cases (24/26), even after ACLR was fixed at 20 N (10 N each). ACLR, anterior cruciate ligament reconstruction; LM, lateral meniscus.

The change in pivot shift acceleration before and after LM repair under provisional fixation of the ACL grafts at 10 N for each bundle (20 N in total) is shown in Figure 2. LM repair reduced pivot shift acceleration in most cases, even after ACLR was fixed at 20 N and pivot shift acceleration decreased significantly from 4.3 ± 1.2 to 2.9 ± 0.9 m/s^2^ with LM repair (*p* < 0.001).

### MRT before and after LM repair

**Figure 3:**
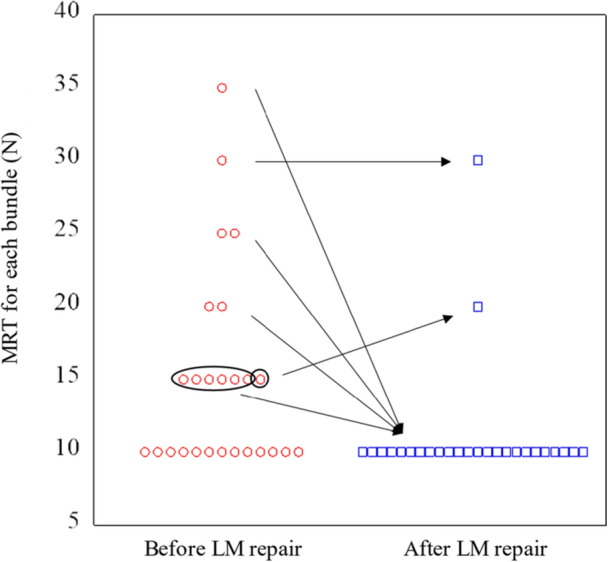
MRT before and after LM repair. The MRT was already 10 N in 13 cases before LM repair. LM repair reduced MRT to 10 N each in 11 of the remaining 13 cases. LM, lateral meniscus; MRT, minimum required tension.

The MRT for pivot shift acceleration was less than that for the healthy side in the pivot shift test before and after LM repair in most cases, as shown in Figure 3. The MRT was 10 N without LM repair in 13 patients (50%). The LM repair reduced the MRT to 10 N each in 11 of the 13 remaining patients. The remaining two patients were not affected by LM repair.

### Factors affecting changes in pivot shift acceleration

Patient characteristics and bone morphology data were used as explanatory variables to investigate factors affecting the change in pivot shift acceleration before and after LM repair. Among the patient and the bony morphological factors, only lateral PTS was significantly correlated with changes in pivot shift acceleration (*r* = 0.598, *p* = 0.001) (Table 2). In contrast, medial condylar width showed a weak negative correlation (*r* = −0.38, *p* = 0.05), suggesting a limited association. Univariate regression analysis indicated that the medial condyle width and lateral PTS reached a *p* value of 0.2. These variables were included in a subsequent multiple regression analysis, which revealed that lateral PTS was significantly associated with the change in pivot shift acceleration before and after LM repair (partial regression coefficient, 0.19; *p* = 0.0043) (Table 3).

**Table 2:** Factors that affect the changes in pivot shift acceleration.

	*r*	95% CI	*p* Value
Age	−0.17	[−0.53; 0.23]	n.s.
BMI	−0.21	[−0.55; 0.20]	n.s.
Hyperextension injured side	0.17	[−0.23; 0.52]	n.s.
Hyperextension healthy side	0.23	[−0.17; 0.57]	n.s.
Knee arthrometer side‐to‐side	0.21	[−0.19; 0.55]	n.s.
Knee arthrometer healthy side	0.14	[−0.26; 0.50]	n.s.
Preoperative acceleration injured side	0.12	[−0.29; 0.48]	n.s.
Preoperative acceleration healthy side	−0.18	[−0.53; 0.23]	n.s.
MM injury	−0.042	[−0.42; 0.35]	n.s.
Medial condyle width	−0.38	[−0.67; 0.0029]	n.s.
Lateral condyle width	−0.097	[−0.40; 0.38]	n.s.
Medial posterior tibial slope	−0.037	[−0.42; 0.36]	n.s.
Lateral posterior tibial slope	0.60	[0.27; 0.80]	0.001
Distal femoral condyle offset	−0.015	[−0.40; 0.37]	n.s.

*Note*: *r* (correlation coefficient) was calculated with Spearman's rank correlation coefficient. Statistically significant difference was defined as *p* < 0.05.

Abbreviations: BMI, body mass index; CI, confidence interval; MM, medial meniscus; n.s., not significant.

**Table 3:** Results of multiple regression analysis for factors associated with changes in pivot‐shift acceleration before and after LM repair.

	*β*	SE	*t*	*p* Value
Medial condyle width	−0.14	0.10	−1.40	n.s.
Lateral posterior tibial slope	0.19	0.060	3.2	0.0043

*Note*: Statistical significance was defined as *p* < 0.05.

Abbreviations: LM, lateral meniscus; n.s., not significant; SE, standard error.

## DISCUSSION

The most important finding of this study was that LM repair contributed to ALRI in most cases, even after ACLR, and that a large lateral PTS was a significant patient factor involved in the LM contribution to control ALRI. Previous studies [7, 8, 11, 24, 32] evaluated ALRI either under ACL‐deficient conditions or after ACLR. Therefore, it remains challenging to determine the respective contributions of the ACL and meniscus to ALRI. This study not only evaluated the actual contribution of LM by measuring ALRI or by adjusting the tension of the ACL graft after ACLR, but also revealed the possibility of reducing the tension of the reconstructed ACL grafts if the LM repair was performed appropriately. In addition, a large lateral PTS was identified as a patient factor in cases showing a large LM contribution to control ALRI. This result suggests that LM repair is crucial in patients with a larger lateral PTS.

In most cases (92.3% for pivot shift acceleration and 84.6% for MRT, except for ACL‐dominant cases), LM repair reduced both tibial acceleration and MRT. This result indicates that the ACL and LM share force during a pivot shift in most cases, and even after ACLR, the LM may play a pivotal role in controlling ALRI. The meniscus is considered a secondary restraint [24, 30], and the importance of LM, in particular, has been reported with respect to stability in the pivotal shift phenomenon [8, 24, 32]. Shybut et al. reported that LMPRT caused additional laxity during the pivot shift phenomenon in knees deficient in ACL, supporting the concept that LM is a secondary restraint in pivot shift loading [32]. Similarly, Frank et al. demonstrated in a biomechanical study using cadaveric knees that LMPRT in ACL‐deficient knees increased the laxity in the pivot shift test and indicated the importance of LM as a stabilizer of ALRI [8]. The results of this study are in line with these reports; even after ACLR, LM repair reduced MRT in most cases. This finding suggests that LM repair can prevent overload of the reconstructed ACL graft. However, LM injuries include not only LMPRT, as highlighted in earlier studies, but also various other tear patterns such as radial, longitudinal and bucket handle tears [6, 19]. The strength and method of repair depend on the condition of the meniscus and the pattern of the injury. Unrepaired LM injuries have been associated with residual ALRI even after ACLR [9]. Therefore, it is important to repair the LM as much as possible, as meniscal repair can restore knee stability to levels comparable to those with an intact meniscus [9]. In the present study, LM repair decreased pivot shift acceleration using various suture techniques in different meniscal injuries. This finding highlights the importance of repairing concomitant LM injuries to preserve function during ACLR.

The present study, which investigated MRT, showed that in half of the cases, ALRI could be controlled using ACL at 20 N (10 N each) before LM repair. Thus, while ACLR accounted for most of the stability of ALRI in some cases, LM also contributed to some extent in other cases. The patient's characteristics and bone morphology were investigated using univariate and multiple linear regression analyses, which identified large lateral PTS as a significant factor. Pearce et al. demonstrated using fresh frozen cadaver knees that ACL graft force increased linearly with greater PTS under axial load and an internal rotation torque [26], indicating that a steep PTS can overload the ACL graft and contribute to ALRI. Furthermore, the STABILITY cross‐sectional study reported that meniscal injury and increased PTS were associated with a preoperative high‐grade pivot shift [1]. Kataoka et al. also reported that a large lateral PTS and greater lateral‐medial slope asymmetry were correlated with a preoperative pivot shift [13]. Similarly, Rahnemai‐Azar et al. noted that ACL‐injured knees with a tibial slope of 9° or greater were predicted to have more rotational laxity [28]. Although a larger PTS is closely related to ALRI, its influence may be accentuated by meniscal injuries. Bernholt et al. reported that large lateral and medial PTS and large lateral–medial slope asymmetry increased the incidence of LMPRT in ACL injuries [2]. Yoshihara et al. also demonstrated that LMPRT was associated with significantly increased lateral‐medial asymmetry of PTS in patients with ACL injuries [36]. Whether the meniscal injury itself or a larger PTS leads to ALRI remains a matter of debate.

This study has several limitations. First, the type of meniscal injury, the location or the number of sutures were not investigated. The degree of meniscal degeneration and the extent of damage could also affect the results. Second, there were variations in the preoperative period. This study included patients who underwent ACLR relatively early after injury to chronic cases that underwent ACLR approximately 5 years after injury. Chronic ACL‐deficient conditions for 6–12 months are at higher risk of high‐grade pivot shifts [1]. Although participants were scheduled to undergo surgery immediately after their visit to the study institution, the influence of chronicity could not be excluded. Third, the bony tunnel positions of ACLR were not evaluated. The location of the tunnel could affect ALRI in ACLR surgery [31, 35, 39]. This study was performed using double‐bundle ACLR. Tunnel apertures were created within the anatomical position using an outside‐in technique. Double‐bundle ACLR has an advantage with respect to controlling ALRI over single‐bundle reconstruction [17, 20], although this limitation did not significantly influence the conclusions. Fourth, although sufficient for comparing pivot shift acceleration before and after LM repair, the sample size was not sufficiently large to evaluate subdivisions into meniscal injury sites, such as LMPRT. In this study, MM was repaired prior to measurement, and its involvement was not considered in the analysis. Although Mouton et al. reported that the MM, particularly ramp lesions, contribute to rotational laxity [22], the present study did not assess the specific patterns of MM injury. However, any MM tears requiring repair were sutured before measurement, and the impact of MM injury showed a low correlation based on the results of the univariate regression analysis. Finally, while identifying a specific cut‐off value for the lateral PTS would be clinically beneficial for surgical decision‐making, the sample size of the present study (*n* = 26) was insufficient to perform a robust statistical analysis (such as a receiver operating characteristic curve) for threshold determination. Future studies with larger cohorts are warranted to establish a reliable cut‐off value.

The clinical relevance of this study is that preservation of the meniscus during ACLR is definitely important, and especially in cases of large PTS, LM repair could reduce stress on the ACL graft by sharing force with the ACL during pivot shift movements. In addition, anterolateral augmentation or anterior closed‐wedge osteotomy should be considered in cases of large PTS when LM repair is difficult to preserve.

## CONCLUSIONS

LM repair decreased pivot shift acceleration and MRT, indicating the importance of LM repair in the force sharing with the ACL. Moreover, the repair of the LM contributed significantly to ALRI in patients with a larger PTS. Therefore, in ACLR, the LM should be repaired whenever possible.

## AUTHOR CONTRIBUTIONS

Masaki Amemiya analysed the data and drafted the manuscript. Yusuke Nakagawa, Tomomasa Nakamura, Nobutake Ozeki, Takashi Hoshino, Mai Katakura and Aritoshi Yoshihara conducted the study and collected the data. Yusuke Nakagawa and Ichiro Sekiya analysed the data. Hideyuki Koga designed the initial plan, conducted the study and completed the final manuscript. All authors read and approved the final manuscript.

## CONFLICT OF INTEREST STATEMENT

The authors declare no conflicts of interest.

## ETHICS STATEMENT

This study was approved by the Institutional Review Board in Tokyo Medical and Dental University (research protocol identification number: M2000‐1566). All study participants provided their full written informed consent for participation in this clinical research prior to the operative procedure.

## Data Availability

Data supporting the findings of this study are available from the corresponding author upon reasonable request.
